# Calvin klein (CK) designer cocktail, new “Speedball” is the “grimm reaper”: Brain dopaminergic surge a potential death sentence

**DOI:** 10.15761/JSIN.1000227

**Published:** 2020-04-24

**Authors:** Mark S Gold, Jean Lud Cadet, David Baron, Rajendra D Badgaiyan, Kenneth Blum

**Affiliations:** 1 Department of Psychiatry, Washington University School of Medicine, St. Louis, Mo, USA; 2 National Institute on Drug Abuse, National Institutes of Health, Bethesda, MD, USA; 3 Graduate School of Biomedical Sciences, Western University Health Sciences, Pomona, CA, USA; 4 Department of Psychiatry, Icahn School of Medicine Mt Sinai, New York, NY, USA; 5 Department of Psychiatry, South Texas Veteran Health Care System, Audie L. Murphy Memorial VA Hospital, San Antonio, TX, Long School of Medicine, University of Texas Medical Center, San Antonio, TX, USA

On June 18^th^ the world lost a fabulous 17-year-old violinist prodigy a Carnegie Hall sensation, Katya Tsukanova, due to a fatal overdose of the designer drug named Calvin Klein (CK). The CK drug is so named because it contains a combination of both cocaine and ketamine. It is therefore appropriately categorized as a “polydrug.” CK (sometimes called CK1 or cable) has become a real party drug not only in England but also in other parts of the world including New York’s nightclub scenes. The feeling experienced from using CK is akin to that induced by the famous sex-inked, Molly, in the 60s and 70s. People taking this drug, mostly by snorting it, suggest that the mixture of the two-produce powerful euphoric highs coupled with a hallucinogenic feeling caused by ecstasy.

In reality, both cocaine [1] and ketamine [2,3] is quite dangerous when taken alone. When taken together, they can therefore cause life-threatening conditions. The clinical use of the anesthetic, ketamine, in veterinarian practices, is associated with high risk for abuse and fatality. Certainly, both cocaine and ketamine have addiction risk [3,4] based on their biochemical effects in the brain. These include blocking of dopamine transporters and increased accumulation of dopamine in mesolimbic and other brain regions [5]. The list of adverse effects of both cocaine and ketamine taken in high doses (as observed as a party drug combination) include violent behaviors, bad trips, psychotic reactions, heart problems, high blood pressure, strokes and other neurological complications, as well as death. Ironically, Calvin Klein himself has experienced an abuse problem but the deadly life-threatening effects causing increased heart rate, palpitations and even cardiac arrest is reminiscent of the cocaine-induced sudden death of star basketball athletes. The NIDA director, Nora Volkow, has warned about the illegal abuse of ketamine now known worldwide for its rapid anti- depressant effects in treatment-resistant depressive (TRD) patients that constitute about 30% of people with clinical depression [6].

Biochemical studies of cocaine have provided evidence for the accumulation of dopamine in various brain areas that contain dopaminergic terminals including the mesolimbic region (nucleus accumbens (NAc) [7]. Repeated high doses of cocaine, by blocking dopamine transporters located in DA terminals [8,9], could result in life-treating catecholamine (dopamine, norepinephrine) levels in peripheral organs such as the heart that might be associated with dysrhythmia and cardiac arrest. It is important to note that the neuropsychopharmacological profile of ketamine and its mechanism of action have been discussed by Blum *et. al.* [10] who report that high doses of ketamine may induce significant accumulation of dopamine in the brain. Of interest, Kokkinou *et. al.* [11] have also proposed a mechanism of action via which ketamine can induce changes in dopamine levels in the brain. Accordingly, ketamine, by blocking NMDA receptors on GABAergic interneurons, could lead to disinhibition of glutamate neurons projecting to dopamine neurons in the midbrain, thereby augmenting glutamate release with consequent enhanced dopamine neuronal firing and increased dopamine levels in the striatum and cortex of rodents. A meta-analysis involving 40 original peer-reviewed studies provided evidence for the role of dopamine in ketamine’s antidepressant effects. In fact, even in several rodent investigations, acute ketamine administration significantly enhanced dopamine levels in the cortex, striatum, and the NAc. Compared to controls, ketamine induced a 62-180% increase in dopamine neuron population firing. With chronic ketamine administration, they found cortical dopamine release to be from 88-180%. Of interest is the fact that sub-anesthetic doses of ketamine in healthy humans, acutely produces symptoms akin to schizophrenic symptoms. Ketamine abusers also can present with psychosis.

While it is true that the DEA currently does not recognize or acknowledge the CK cocktail as a specific polydrug type, the agency does reference cocaine and ketamine in their drug schedule. Nevertheless, it is important that educational efforts are spent to educate the public about the cause of death of young people like Katya Tsukanova. These young partying teens, seeking new highs or feelings, may not have any idea about the medical dangers and/or lethality of CK and other similar psychoactive drugs. This dangerous situation requires a concerted effort from governmental agencies and media outlets. Better education of parents and teens are also needed. However, changing the name of CK to the “Grimm Reaper” may stimulate the curiosity of some young adults who might want to experiment with this dangerous combination. Instead, it is more important to reach out to other professionals in order to develop more sensible preventive and treatment approaches that encompass changes NOW!
